# Colloid Metals in the Clinical Laboratory

**Published:** 1914-06-13

**Authors:** 


### Colloid Metals in the Clinical Laboratory.

The use of colloid metals in therapeutics was reviewed in a special article in a recent issue of The Hospital (May 2, 1914, p. 125); but nothing was said of them as reagents in the clinical laboratory. Two workers at the Johns Hopkins Hospital, Baltimore—Drs. S. R. Miller and R. L. Levy—contribute to the *Bulletin* of that institution an account of their researches into colloid gold as it affects the cerebro-spinal fluid. In 1912 Lange described a method whereby a differentiation could be thus made between syphilitic and non-syphilitic affections depending on a precipitation of protein from the cerebro-spinal fluid in certain dilutions by means of colloid gold. Miller and Levy have extended the scope of the test, and formulate some definite conclusions which seem to indicate that at present it is not of much practical use, but that the underlying principle may turn out to be of very great value. The colloidal gold test, they remark, is essentially a laboratory method. It can be performed rapidly and with a minimal amount of spinal fluid. Extreme care in the preparation of reagents and the cleaning of glassware is imperative. Normal fluids give negative reactions. The test is of no aid in the diagnosis of purulent or tuberculous meningitis. It has no advantages over known laboratory methods in the diagnosis of congenital syphilis. Reactions in secondary and tertiary syphilis are inconstant, and their significance when present is doubtful. There is no proof that a positive reaction means the involvement of the central nervous system. The positive reactions in the majority of cases of tabes and cerebro-spinal syphilis are not characteristic; but the reaction peculiar to paresis is sufficiently constant to warrant its use as an aid in the differentiation of this condition from others with which it might be confused. It is possible that the test may prove to be more sensitive than those which are in use at present.

